# Cohabitation and Mortality Across the Life Course: A Longitudinal Cohort Study with Swedish Register-Based Sibling Comparisons

**DOI:** 10.1007/s10680-024-09722-6

**Published:** 2025-01-14

**Authors:** Jesper Lindmarker, Martin Kolk, Sven Drefahl

**Affiliations:** 1 Institute of Analytical Sociology, Linköping University, Linköping, Sweden; 2 Stockholm University Demography Unit, Department of Sociology, Stockholm University, Stockholm, Sweden; 3 Institute For Futures Studies, Stockholm, Sweden; 4 Faculty of Education and Welfare Studies, Åbo Akademi University, Vasa, Finland

**Keywords:** Cohabitation, Register data, Sweden, Mortality, Civil status

## Abstract

Research has shown that married individuals live longer lives than unmarried women and men. A smaller number of studies have included non-marital cohabitation and have found that their mortality falls between the married and other unmarried groups. There are indications that the cohabiting population is diverse in terms of mortality risk, yet very little is known about how the association is related to age and stages of the life course. Sweden is a forerunner in family trends, and this is the first study that examines cohabitation and mortality in a Swedish context. Using Swedish register data for the years 2012–2017, we investigated how different partnership statuses are related to mortality for men and women at different ages (*N* = 5,572,011). We also examine whether the association between cohabitation and mortality is similar after accounting for family-of-origin effects through the use of a sibling comparison design. Our findings confirmed the notion of cohabiters as a diverse group whose relative mortality risk differs depending on the timing of cohabitation. Never-married cohabiters had a mortality risk similar to married couples at younger ages and a gradually increased risk with age. Divorced and widowed cohabiters exhibited an age gradient in the opposite direction. Future research should consider how the context of cohabitation changes across the life course.

## Introduction

Being married has consistently been found to be associated with a longer life (Lillard & Panis, 1996; Murphy et al., 2007; Dupre et al., 2009; Rendall et al., 2011). The proposed mechanisms are divided between selection explanations and those focusing on social causation. Selection mechanisms refer to the various ways in which healthier individuals are more likely to marry and remain married, as well as the influence of factors that simultaneously affect both health and the likelihood of marrying. Social causation mechanisms, on the other hand, refer to how being in a marriage promotes healthier behaviors, enables the pooling of resources, and provides spousal support, all of which contribute to lower mortality (Drefahl, 2012). A question that has not been thoroughly answered is to what extent the above-mentioned protective effect of partnering applies similarly to cohabiting couples. The research conducted to date suggests that cohabiters show better health across multiple measures than do non-partnered individuals; however, their health is not on par with that of married individuals (Carr & Springer, 2010).

Research on cohabitation has become increasingly important, as cohabitation as a partnership status became more common in societies with progressive social norms throughout the late 20th century. For societies where cohabitation is widespread, it first began as a prelude to marriage and was seen as a short phase in the transition into adulthood (Billari & Liefbroer, 2010). However, over time cohabitation has increasingly been adopted as a long-term alternative to marriage (Brown Bulanda & Lee, 2012). Sweden was early to adopt cohabitation practices (Ohlsson-Wijk et al., 2020; Sobotka & Toulemon, 2008), and it is the European country in which the second-largest share of first births take place in cohabiting unions (Andersson et al., 2017), after Iceland (Jónsson, 2021), making it an ideal context to examine the association between cohabitation and mortality.

The trends of increasing adoption of cohabiting raise questions about whether the protective effects typically associated with marriage also apply to those who are cohabiting. Additionally, increasing trends of long-term cohabitation and cohabitation at older ages (Brown & Wright, 2017) emphasizes the need to examine how these associations change across life stages. The aim of this study was to gain a more detailed understanding of the association between partnership status and mortality; specifically, it examined the heterogeneity of cohabiters, differentiating between those who are cohabiting without having ever been married and those who cohabit after one or more marriages, across age groups to represent variations of stages in the life course. This research presents a number of innovations in the study of the association between mortality and partnership status. Using rich longitudinal individual data of the total Swedish population, we were able to make use of marital histories and examine mortality differences by partnership status while controlling for a number of background characteristics. We also used sibling fixed effects to adjust for unobserved social background characteristics and ensure that any observed effects of partnership histories and mortality are not due to confounding with family of origin.

## Literature Review

### Partnership Status and Mortality

Empirical studies across varied time periods and societies have consistently found that married individuals experience considerably lower mortality rates than those who are single, divorced, or widowed (Dupre et al., 2009; Roelfs et al., 2011; Rendall et al., 2011; Manzoli et al., 2007). The effects are usually more pronounced among men than women (Staehelin et al., 2012). While some variations have been noted, depending on the specific cause of death, a consistent pattern of reduced mortality being associated with marriage prevails (Ben-Shlomo et al., 1993). Scholars have suggested that these observed disparities arise from both the positive health effects of marriage and the higher propensity for individuals with poorer health to divorce or remain single (Hu Goldman, 1990; Lillard & Panis, 1996; Waldron et al., 1996).

As an extension of earlier work on marital status and mortality, researchers have begun to examine how the beneficial impact on mortality extends to partnerships more generally. Highlighting the nuanced relationship between partnership and mortality, studies have found that the mortality rates among cohabiting individuals generally fall between those of married and those of unmarried individuals (Koskinen et al., 2007; Drefahl, 2012; Staehelin et al., 2012; Franke & Kulu, 2018).

Koskinen et al. (2007) examined this dynamic within the context of Finland and found an excess mortality rate of 66% among cohabiting men and women of working age compared to their married counterparts. However, the contrast was less stark in the 65+ group, where excess mortality rates of 41% for men and 36% for women were reported. Meanwhile, a study focusing exclusively on older adults in Italy yielded different results (Scafato et al., 2008). They found significant mortality differences between married and cohabiting men, whereas no such difference was found among women. Further, a Swiss study by Staehelin et al. (2012) concluded that cohabitating men and women show comparable excess mortality compared to the married in all age groups and that the only gender differences arose from how men were more affected by living alone. An association between partnership status and cause-specific mortality was found for men in Sweden, but not for women. This association was significant for all-cause, cardiovascular, and other causes of mortality, but not for cancer (Lindström et al., 2024). These disparate findings underscore the conclusion that the impact of partnership status on mortality can vary significantly across demographic and cultural contexts.

The proposed mechanisms for the lower mortality of those with partners are divided between selection effects and protection effects. However, the consensus is that it is less of an either-or discussion and rather a question of how much each mechanism contributes to the total (Carr & Springer, 2010). Selection effects encompass two aspects: health selection and social selection. Health selection refers to underlying health factors that impact an individual’s likelihood of entering a partnership. While there is plenty of support for positive selection, with healthier individuals more often forming marriages (Goldman, 2015), there is also evidence suggesting adverse selection, where individuals with poorer health tend to remarry more quickly (Lillard & Panis, 1996). Similarly, there is a selection effect out of marriage, meaning that healthier individuals are less likely to transition out of marriage via divorce or widowhood. For example, it has been shown that married couples who are faced with morbidity or disability are more likely to separate (Wyke & Ford, 1992).

The second type of selection, social selection, encompasses confounding social factors that both influence mortality risk and if individuals manage to form or maintain partnerships. Such factors can include education, social class, or income, along with personality traits and other individual characteristics and habits. For example, the social gradient of marriage in Scandinavia has become increasingly pronounced, with individuals of higher educational attainment and income being more likely to form partnerships and less likely to remain childless or unpartnered (Hoem, 1997; Ohlsson-Wijk, 2011; Kolk & Barclay, 2021; Chudnovskaya, 2019). Then, given the known association between higher socioeconomic status (SES) and lower mortality, we anticipate that married and cohabiting individuals will have lower mortality than unpartnered women and men. The association may be particularly pronounced for cohabiters in younger cohorts due to the reversal in the social gradient, from negative to positive, between cohabitation and education in the 20th century (Perelli-Harris et al., 2010). In sum, these aspects necessitate rigorous controls for both adult and family background SES in studies investigating the link between mortality and partnership status.

It is also important to note that the selection dynamics related to SES not only determine the propensity to find and maintain a partnership but may also drive significant differences in mortality rates between married and cohabiting individuals across different SES levels. This is illustrated by a study using Danish register data, which found that when controlling for indicators of SES, the mortality differences by living arrangement decreased more significantly for men than for women (Drefahl, 2012). Additionally, the study identified a mortality crossover in relation to partnership status and SES interaction, showing that cohabiting men and women with low SES experienced higher mortality compared to their married peers. Conversely, those with high SES had lower mortality than their married counterparts (Drefahl, 2012).

Another potentially relevant aspect is the size of the group in question. In countries where a smaller share of the population remains unmarried or divorced, their respective mortality risks are thought to be higher (Hu Goldman, 1990), suggesting a potential stigma associated with being part of a less common group, or alternatively, a concentration of characteristics associated with higher mortality. Thus, the difference in mortality rates between married and cohabiting individuals might be larger in contexts where cohabitation is less common or considered non-normative. Conversely, in societies where cohabitation has become a mainstream lifestyle choice, one might expect a smaller mortality difference between cohabiters and married individuals.

Protection effects, or social causation effects, encompass several mechanisms. First, married couples tend to experience more economic stability which has positive health effects(Carr & Springer, 2010). In Sweden, married individuals must actively opt out of combining their finances, whereas for cohabiters, the default is to keep financial assets separate. Pooling assets can enhance the efficiency and stability of resources for both partners in a relationship. This is more pronounced for women, who generally have lower incomes (Drefahl, 2012). Second, married individuals tend to have healthier behaviors, which is usually attributed to social control exerted over each other (Umberson, 1987). This involves fewer unhealthy habits, such as bad food habits and smoking, as well as less risk-taking. Social control effects are stronger for men, as women tend to monitor their partners to a larger degree (Carr & Springer, 2010). Lastly, spouses provide support and care to each other, both emotional and instrumental, which decreases stress and has generally positive consequences for well-being (Rendall et al., 2011). Partners may also often monitor each other’s health. This is particularly relevant at older ages, since the spouse is the most common informal caregiver, which mostly manifests as wives caring for their husbands (Agree & Glaser, 2009). Given that the above protection mechanisms are gendered, if they were more or less prevalent in cohabiting unions than in marriages, we would expect to find gender differences in the mortality of cohabiters.

Selection and protection effects also apply to individuals who have experienced union dissolution or the loss of a partner. These events, such as divorce or widowhood, are significant life stressors that can lead to similar adverse health outcomes, setting these individuals apart from those who have never partnered. Remarriage or repartnering through cohabitation adds further complexity, as the stability and quality of these new unions can either alleviate or intensify the effects of the original stressor (Grundy & Tomassini, 2010).

Numerous individual characteristics may influence the association between partnership status and mortality, affecting both the protective effects and the selection mechanisms to varying degrees across the life course. A critical factor to consider is the presence of children, particularly the condition of childlessness (Dykstra, 2009). Childbearing decisions are inherently tied to partnership status making it an essential component in the relationship between partnership status and mortality. At younger ages, childlessness could capture health selection associated with infertility or social selection mechanisms unrelated to SES, such as risky or unhealthy behaviors that deter individuals from forming partnerships or having children. Moreover, younger parents are more likely to have their children residing in the household, which exerts a significant influence on parents’ behaviors in comparison to older parents, whose children have mostly moved out of the household (Drefahl & Mussino, 2023). In later stages of life, children are crucial for maintaining adequate levels of instrumental and emotional support, and childlessness may signify the absence of such support (Agree & Glaser, 2009).

A common perspective suggests that cohabitation offers fewer benefits compared to marriage, characterized by poorer union satisfaction, greater instability, and poorer well-being outcomes. However, some research suggests that these associations are weakened or eliminated after accounting for selection into cohabitation or marriage (Sassler & Lichter, 2020). Further, the literature underscores the heterogeneity of cohabiting individuals and shows varying mortality differences across stratum such as SES and age (Carr & Springer, 2010). Such variability is perhaps unsurprising considering that partnership statuses, including cohabitation, are influenced by, and influence, multiple facets of an individual’s life, such as socioeconomic status, the decision to have children, and societal norms surrounding partnership and cohabitation. The manifestation of these selection and protection effects are a complex interplay of social, personal, and contextual factors which may lead to many different patterns of mortality differences between married and cohabiting individuals.

### The Rise of Cohabitation

Having discussed the complex interplay between personal, social, and contextual factors in determining the association between partnership status and mortality, it is crucial to consider the historical transformation that has reshaped family norms and structures. In the second half of the 20th century, family patterns in many Western societies began undergoing substantive changes, a transformation that has been referred to as the second demographic transition (Lesthaeghe, 1995). A defining feature of this transition is a shift in the nature of family formation: where marriage was once nearly universal, cohabitation has become increasingly prevalent, and often in some groups replace marriage completely.

These trends are evident when examining the first unions of European women. Among those born between 1930 and 1939, few started their first union as unmarried cohabiters, with proportions ranging from close to 0% in Spain to 21% in Sweden. Contrast this with women born between 1970 and 1979: 38.3% in Spain and 93.3% in Sweden started their first union as cohabitation (Billari & Liefbroer, 2010). The rising prevalence of non-marital births, which serve as an indicator of cohabiting parenthood, underscores this shift. In Sweden, non-marital births constituted 11% of the total births in 1960, but by 2000, this figure had risen to 55% (Thomson et al., 2019). While these trends are most pronounced in Northern Europe, they are also observable in Western Europe and, albeit to a lesser extent, in Southern Europe (Billari & Liefbroer, 2010).

The progression of cohabitation and its relationship to marriage depends on the context and manifests in ideal types, through which societies progress (Heuveline & Timberlake, 2004). Importantly, not all countries follow this pattern, and most studies focus primarily on European or Western countries, casting at least some doubt on the universality of the findings. Typically the historic change begins when cohabitation becomes a viable choice for young adults who want to initiate their union as cohabiters before transitioning to marriage; cohabitation effectively functions as a trial marriage. Then, as cohabitation gains wider acceptance by society, it becomes the norm for a first union and the cohabiting phase is extended. Ultimately, cohabitation gains acceptance as a comparable alternative to marriage and as a suitable arrangement for childbearing (Sobotka & Toulemon, 2008). This final stage is where Sweden finds itself today (Ohlsson-Wijk et al., 2020).

The historical transformation is particularly evident in Sweden, which has a history of and reputation for being a family forerunner (Ohlsson-Wijk et al., 2020). In terms of demographic trends, Sweden is consistently among the first, if not the first, to begin shifts. This has been shown for postponement of marriage, increasing divorce trends, family complexity, and lately, the stabilization of divorce rates and increasing marriage rates (Sobotka & Toulemon, 2008; Thomson, 2014; Sobotka, 2008; Ohlsson-Wijk et al., 2020). Furthermore, Sweden is an outlier in terms of having more progressive and secular values than any other country (World Values Survey, 2020). For example, factors that are considered important drivers of progressive demographic trends in family formation, such as egalitarian attitudes and public policies enabling gender equality, are widespread in Sweden (Oláh & Bernhardt, 2008).

In a European comparative study, Sweden was the only country in which the majority of births occur with the mother in a cohabiting union, and where individuals spend the highest shares of their lives in a cohabiting union, both with and without children (Andersson et al., 2017). In Appendix Fig. 2, we show civil status trends in Sweden from 1970 to 2020, illustrating the large share of the never-married and the corresponding decline in marriage following increasing cohabitation in Sweden since the 1970s. Despite the high prevalence of cohabitation in Sweden, marriage has seen a resurgence since the end of the 1990s (Ohlsson-Wijk et al., 2020), and over 50% of cohabiting unions that have not dissolved transition into marriage within 10 years of their formation (Andersson et al., 2017).

Patterns of partnership formation differ significantly over the life course. At younger ages, partnering is generally a question of “when,” while at older ages it becomes a question of “if,” as interest in, and the likelihood of, forming an intimate union consistently decreases with age (Brown Bulanda & Lee, 2012). The objectives of and context around cohabitation also vary: younger couples commonly view partnership as the foundation for family formation and raising children, whereas older couples tend to have previous partnership experiences and may have children from those unions (Rapp, 2018). Consequently, entering a new partnership in the later stages of life necessitates a framework that accommodates both the new partner and the children or partners from previous partnerships. In line with trends at younger ages, cohabitation is increasingly seen as a long-term alternative to marriage at older ages (Vespa, 2012; Wright, 2020). Older cohabiters are found to stay longer in the union, report higher union quality, and less often have plans for marriage than their younger counterparts (Brown & Wright, 2017).

In the context of cohabitation and mortality, no study has comprehensively examined life stages, and only a handful have analyzed age and partner histories (Koskinen et al., 2007; Staehelin et al., 2012; Franke & Kulu, 2018). These studies suggest that the difference in mortality rates between cohabiters who are divorced or widowed and those who have never been married is much larger at younger ages than at older ages. This implies that older cohabiters who are divorced or widowed may not be as negatively selected, which may be linked to the finding that older couples often favor cohabitation over marriage, as it offers fewer obligations and more flexibility for those with children from earlier unions (Brown et al., 2019). In contrast, young cohabiters who are divorced or widowed appear to be more negatively selected, as they possess some characteristics that led them to experience divorce or widowhood at a young age. Thus, considering the evolving social landscape of family formation and the diverse contexts and motivations related to cohabitation at different life stages, it becomes crucial to account for these variations when analyzing the relationship between cohabitation and mortality.

## Research Question

The objective of this study was to investigate the relationship between partnership status and mortality across life stages. Specifically, we aimed to understand how the association between non-marital cohabitation and mortality differs among those who cohabit before marriage and those who cohabit after being married at least once. This study provides the first evidence of the association between partnership status and mortality in Sweden and fills a gap in the existing literature regarding how this association varies by age group.

Based on studies conducted in other contexts, we hypothesized that cohabiters would have higher mortality than married individuals. However, given Sweden’s broad acceptance of cohabitation and its prevalence, we anticipated that the differences between married individuals and cohabiters would be less pronounced than had been documented in other studies. Also, cohabiters who had never been married would likely share many characteristics and protective mechanisms with married individuals, given that most of them eventually transition into marriage. Consequently, we assumed that excess mortality among never-married cohabiters would be significantly less pronounced than among cohabiters who had previously been married.

We further expected the size of the differences to vary by age group. They could increase to the extent that advantages either accumulate or change character depending on age. For example, given the increasing importance of support from a partner with age, mortality differences should increase if there are differences in the quality of support between partner statuses. In terms of selection, the impact of positive and negative selection with respect to mortality on entering and exiting certain partnership statuses could change with age. For example, being divorced or widowed at a younger age is relatively rare and is associated with a number of negative mortality-related characteristics, leading to significantly higher excess mortality than at later ages, regardless of cohabitation status. Conversely, while excess mortality among never-married cohabiters is expected to be low at younger ages, as a large share of individuals transition into marriage, the remaining individuals possibly share negative health characteristics which would increase excess mortality with age. Further, for all partner statuses, as the frailest members die we would expect mortality rates to converge between groups with higher age (Goldman, 2001). Thus, we generally expect relative mortality differences to decrease with age.

An important contribution of the current study is the application of a sibling design. By comparing full siblings, who have most likely shared an early life course, it is possible to account for factors shared by siblings, such as parental education, geography in childhood, schooling, ethnicity, and many factors that are hard to measure, such as parental values, socialization, and religiosity. This approach has not previously been used to study partner status and mortality. We expected that some of the mortality differences between cohabiters and married individuals could be explained by unobserved characteristics attributed to upbringing. When only comparing siblings that share certain background factors (e.g., religious background, parental values, family SES, and thus adjusting for such factors) in our sibling fixed effect models, we expected to see further attenuation of any differences.

## Data

For this study, we used Swedish register data to analyze the association between partnership status and mortality. The research was approved by the Swedish National Ethical Review Board (nr: 2017/1623-31/5). The study period was 1 January 2012 to 31 December 2017. The inclusion criteria were being born in Sweden and having been registered in Sweden in 2011. 2011 was the first year with reliable dwelling register data, which allowed us to include cohabiting unions. A further criterion was that an individual was 30 years old or older on 1 January 2012 or turned 30 between 1 January 2012 and 31 December 2017. To exit the population, there were two possible events: death between 1 January 2012 and 31 December 2017 or emigration from Sweden between 1 January 2012 and 31 December 2017. This resulted in a study population of 5,572,011 individuals who had been in the population for at least one year.

Data on marital status were obtained from the registers on civil status. Data on non-marital cohabitation were extracted from the dwelling register, which uses a heuristic to infer a cohabiting union when two non-married adult individuals of different sex, not closely related, with less than 15 years of age difference, live in the same dwelling (Statistics, 2017). The methodology employed by Statistics Sweden aligns with established approaches in other Nordic countries, such as Denmark (Drefahl et al., 2012) and Finland (Koskinen et al., 2007). This heuristic is likely more inaccurate for younger individuals, who commonly have temporary living arrangements, such as shared apartments during their education. Additionally, mortality rates at these ages are very low, which is why we decided to exclude individuals younger than 30 from all our analyses. Our study concerns the difference between non-marital cohabiting unions and marriages; thus, an individual can be either classified as cohabiting or married, not both. Married individuals could either be living with their partner or not and still be classified as married.

The variable *partnership status* (time-varying) was prepared by combining marriage status with data on cohabitation into the following categories: “Married,” “Never married, in a cohabiting union,” “Never married, not in a cohabiting union,” “Divorced/Widowed, in a cohabiting union,” “Divorced, not in a cohabiting union,” and “Widowed, not in a cohabiting union.” Those whose partnership status was not consistent across registers or otherwise unknown were classified as “Other.”

We recognize the importance of distinguishing between first and subsequent marriages, as this distinction is particularly relevant when comparing previously married cohabiters. However, our data is left-truncated, meaning we lack complete marriage histories for all cohorts. This limitation makes it unreliable to determine whether a current marriage is a first or subsequent one.

We controlled for three variables known to affect the association between partnership status and mortality. *Childless* was a time-varying covariate indicating whether the individual had a living child or not. Education (time-varying) was coded as the highest level of education and divided into five categories: (1) elementary education, (2) upper secondary education, (3) some tertiary (less than 2 years) or other supplementary education, (4) more than two years of tertiary education, and (5) doctoral education. Income quartiles (time-varying) were based on disposable income and calculated by the cohort of the Swedish-born population each year of analysis.

The proportion of missing information in the data can be considered low, as it was consistently below 1%. Missing values on partnership status were coded as “Other” and included in the analysis; for childlessness, there were no missing values; for SES variables, missing values were coded as a separate category and included in the analysis.

## Plan of Analysis

For our analyses, we used event history analysis (survival analysis). We used the Cox proportional hazards model, which is defined as:

$$h_{i}(t) = h_{0}(t)\text {exp}(\beta _{1}x_{i1} + \beta _{2}x_{i2} + \ldots + \beta _{k}x_{ik}),$$

where $h_{i}(t)$ is the individual hazard rate at time t and $h_{0}(t)$ is the baseline hazard at time t, which represents the hazard when both of the independent variables $x_{1} \text { and } \ldots x_{k}$ are equal to zero. $\beta _{1},\beta _{2} \ldots \beta _{k}$ are the estimated coefficients of the model.

The Cox proportional hazards model (Cox, 1972) is a nonparametric model that makes no assumptions about the shape of the baseline hazard, which means that conclusions can only be drawn about the relative risks between groups. In all models, age is the process time and is thus adjusted for within each age group. To ensure robust standard errors, observations are clustered by individual ID, reflecting multiple entries per subject as values of time-varying covariates change across different years. Furthermore, we tested for the proportional hazards assumption. Mortality was measured with monthly precision, whereas the other covariates were based on yearly changes.

All regressions were calculated separately by sex and three age groups: 30–49, 50–64, and 65+. These are common cutoffs in the literature (Koskinen et al., 2007; Franke & Kulu, 2018) and provide comparisons between young and old age groups while ensuring an adequate number of deaths in each category. The reference category was married individuals. Model 1 included only the covariate partnership status. In Model 2, we added control variables for being childless, highest level of education, and income quantile.

Model 3 is our sibling comparison model. We used the same model equation as above, stratified by sibling group (Barclay et al., 2016), which changed the baseline hazard to $h_{0j}$, allowing it to vary between sibling group strata $j$ (Allison, 2009). Sibling groups were based on the unique full-sibling groups in our data, identified through the Swedish multigenerational register. A consequence of using a sibling comparison is that only individuals with same-sex siblings where at least one sibling died were entered into the analysis. Such a research design can account for confounding factors that affect both mortality and decisions related to choosing certain life pathways, such as cohabitation. Further, we could account for family-of-origin factors in more detail than is possible with observable characteristics of parents, such as parental education, and the design also captures aspects of origin socioeconomic status (SES) shared between siblings that are not well captured by observable register information. Finally, a sibling design accounts for family values, traits, and preferences that may affect both selection into partnership and mortality, such as risk aversion and other family-specific personality traits.

While the Cox proportional hazards model allows us to examine the association between mortality risk and partnership status, one needs to be mindful of causation in this context. Notably, our controls (childlessness, education, and income) are time-varying and could act as both determinants and outcomes of partnership status. For example, being in a union can influence one’s income over time, while simultaneously, income can affect the likelihood of finding a partner and maintaining the union. Similarly, children are a common outcome of being in a union; however, the presence of children could also serve as a determinant of entering a union. The possibility of bidirectional causality presents challenges in disentangling whether partnership status is a cause or consequence of these factors. The sibling comparison is an attempt to tackle some of these issues by accounting for shared background factors; however, reverse causality should be considered when interpreting the results.

To make sure that the results we present from the sibling comparison models were not due to the changing sample properties, in our supplementary materials we present between-sibling models (without sibling fixed effects) with the same population as our sibling comparison models (see Appendix 1).

Additional robustness checks of our models included a model with both genders in the population and an interaction term for gender and partnership status that allowed us to formally compare gender (see Appendix 1. The model confirmed the gender differences from our other models without the interaction term.

Models were estimated using the *stcox* command in Stata version 18.0.

## Results

Tables 1 and 2 show the distribution of time at risk, measured in years, by age group, and sex during the period 2012–2017. All categories have deaths (events) during the study period. The few deaths for those widowed and not cohabiting in the youngest age group should be noted; however, this should not affect the analysis, given the size of the study population. Married individuals contributed the most person-years across all age groups. Notably, there were gender differences, with women more commonly married in the youngest age group and men in the oldest. Men consistently had a larger share in the never-married category, whereas women were more commonly divorced and widowed.

Never-married cohabiters in the youngest age group contributed a quarter of the person-years at risk, while only a few percent in the oldest group. Divorced/widowed cohabiters contributed a small share of person-years at risk in the youngest age group: 3.2% of women and 2.4% of men. These shares were 6.1% and 5.7% in the 50–64 age group, and in the oldest age group, 4.4% for women and 5.7% for men.

**Table 1:** Person years at risk (PY) and deaths for Swedish born women aged 30+ in Sweden, 2012–2017

	Ages 30–49			Ages 50–64			Ages 65+		
	PY	%	Deaths	PY	%	Deaths	PY	%	Deaths
Partnership status									
Married	3 040 902	46,2	1 240	2 731 891	54.3	6 065	2 644 662	42.5	41 420
Never married, not cohabiting	1 214 643	18.5	1 430	648 990	12.9	3 410	387 905	6.2	15 787
Divorced, not cohabiting	429 200	6.5	482	712 044	14.2	2 972	869 343	14.0	28 846
Widowed, not cohabiting	15 287	0.2	28	122 336	2.4	546	1 851 949	29.7	127 900
Never married, cohabiting	1 570 514	23.9	613	464 743	9.2	1 179	93 853	1.5	1 469
Divorced/widowed, cohabiting	211 350	3.2	149	305 911	6.1	857	274 353	4.4	5 277
Other	97 129	1.5	71	42 594	0.8	141	107 945	1.7	5 536
Childless									
No	5 339 604	81.2	2 568	4 334 964	86.2	11 585	5 419 091	87.0	187 639
Yes	1 239 421	18.8	1 445	693 546	13.8	3 585	810 918	13.0	38 596
Highest level of education									
Elementary or lower	370 121	5.6	701	624 384	12.4	3 496	2 393 240	38.4	129 078
Upper secondary	2 752 433	41.8	1 938	2 469 991	49.1	7 829	2 404 818	38.6	68 842
Higher education less than 2 years	399 593	6.1	235	210 700	4.2	472	90 376	1.5	1 426
Higher education at least 2 years	2 961 895	45.0	1 037	1 671 924	33.2	3 177	1 285 351	20.6	23 346
Doctoral education	72 349	1.1	18	43 813	0.9	49	28 952	0.5	399
Missing	22 633	0.3	84	7 698	0.2	147	27 272	0.4	3 144
Income quantile									
0–25	2 163 240	32.9	1 548	1 575 748	31.3	7 846	2 143 199	34.4	72 326
26–50	1 964 461	29.9	1 240	1 537 218	30.6	3 853	1 847 984	29.7	74 599
51–75	1 348 114	20.5	777	1 077 503	21.4	2 073	1 222 594	19.6	45 372
76–100	1 100 053	16.7	438	837 343	16.7	1 398	1 016 118	16.3	33 938
Missing	3 157	0.0	0	697	0.0	0	114	0.0	0
Total over all categories of each variable	6 579 025	100.0	4 003	5 028 509	100.0	15 170	6 230 009	100.0	226 235

Percentages may not sum up to 100 due to rounding

*Source:* Authors’ calculations based on Swedish register data

**Table 2:** Person years at risk (PY) and deaths for Swedish born men aged 30+ in Sweden, 2012–2017

	Ages 30–49			Ages 50–64			Ages 65+		
	PY	%	Deaths	PY	%	Deaths	PY	%	Deaths
Partnership status									
Married	2 831 955	40.8	1 397	2 720 331	52.8	7 262	3 224 244	60.9	90 129
Never married, not cohabiting	1 792 536	25.8	3 763	907 796	17.6	7 946	457 771	8.6	22 059
Divorced, not cohabiting	313 255	4.5	648	577 580	11.2	4 381	551 547	10.4	23 933
Widowed, not cohabiting	6 847	0.1	11	44 404	0.9	282	507 523	9.6	44 200
Never married, cohabiting	1 736 915	25.0	863	563 603	10.9	1 647	160 841	3.0	3 559
Divorced/Widowed, cohabiting	168 173	2.4	158	292 907	5.7	946	299 467	5.7	8 261
Other	90 833	1.3	84	47 288	0.9	255	93 783	1.8	8 161
Childless									
No	4 839 189	69.7	3 535	4 083 317	79.2	14 858	4 408 529	83.3	159 010
Yes	2 101 325	30.3	3 389	1 070 592	20.8	7 861	886 647	16.7	41 292
Highest level of education									
Elementary or lower	653 795	9.4	1 665	1 029 950	20.0	6 801	2 015 033	38.1	100 340
Upper secondary	3 563 392	51.3	3 796	2 532 694	49.1	11 481	2 036 936	38.5	67 742
Higher education less than 2 years	530 298	7.6	405	455 814	8.8	1 333	163 463	3.1	3 127
Higher education at least 2 years	2 067 772	29.8	891	1 044 408	20.3	2 712	976 007	18.4	25 544
Doctoral education	94 056	1.4	48	77 220	1.5	141	81 541	1.5	1 714
Missing	31 200	0.4	119	13 823	0.3	251	22 197	0.4	1 835
Income quantile									
0–25	1 218 632	17.6	2 711	972 251	18.9	9 688	738 228	13.9	32 933
26–50	1 413 158	20.4	1 760	1 006 634	19.5	4 713	1 040 451	19.6	45 957
51–75	2 028 680	29.2	1 329	1 467 062	28.5	4 496	1 657 076	31.3	60 416
76–100	2 276 088	32.8	1 124	1 706 706	33.1	3 822	1 859 128	35.1	60 996
Missing	3 955	0.1	0	1 255	0.0	0	293	0.0	0
Total over all categories of each variable	6 940 513	100.0	6 924	5 153 909	100.0	22 719	5 295 176	100.0	200 302

Percentages may not sum up to 100 due to rounding

*Source:* Authors’ calculations based on Swedish register data

Tables 3 and 4 show the results from our regression models (full regression tables are found in Appendix 1). Model 1 is unadjusted, with partnership status as the only covariate. As expected, married individuals had the lowest mortality at all ages and cohabiters had a mortality between those who married and those who never married, divorced, or widowed and were not cohabiting. Coefficients were significant, however, with high standard errors for the groups with few deaths. On the one hand, those in a cohabiting union without having been married previously experienced only a slightly higher mortality risk than those married in the youngest age group. The difference increased for each subsequent age group. On the other hand, those in a cohabiting union after a dissolved marriage experienced higher excess mortality in the youngest age group than did those in each subsequent age group.

**Fig. 1:**
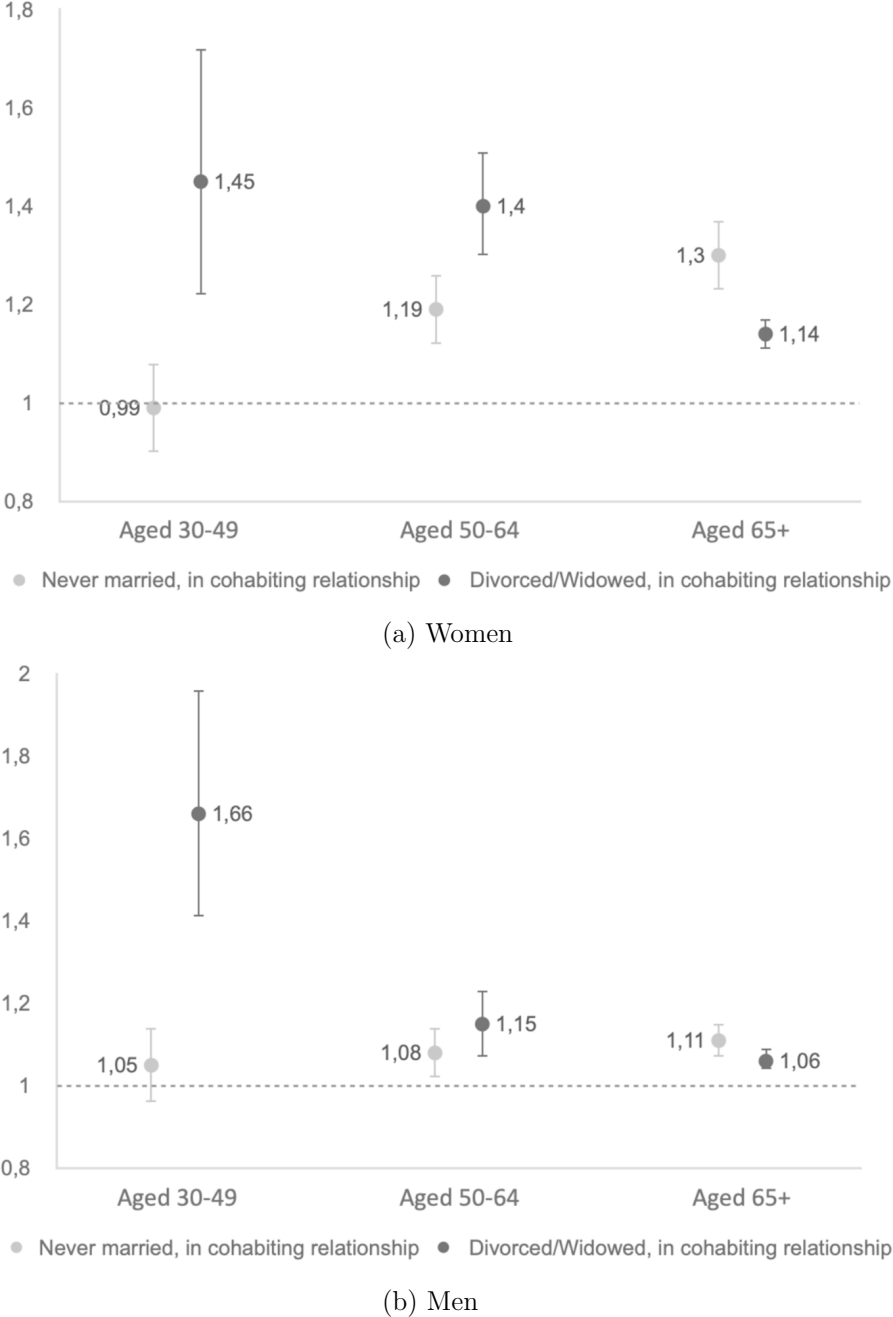
Mortality risk of cohabiters relative to married individuals (reference line). Coefficients from cox regression. Swedish born population aged 30 and above living in Sweden during the period 2012–2017. Analysis controlled for other partnerships statuses, age, childlessness, level of education, and income quantile

In Model 2, we included controls for childlessness, level of education, and income quartile. As expected, these explained some of the differences between groups and thus attenuated the risks for the non-married groups. These results are highlighted in Fig. 1. For both women and men, the mortality risk for never-married cohabiters aged 30–49 was not significantly different from their married counterparts. This was not the case in the unadjusted model (Model 1), suggesting that excess mortality was explained in Model 2 by childless individuals and those with low SES being more likely to cohabit. For the two older age groups, women experienced a sharper rise in relative mortality risk with age compared to men.

Divorced/widowed cohabiters faced a mortality risk gradient that was reversed compared to never-married cohabiters, with the highest excess mortality observed in the youngest age group and each succeeding age group experiencing a reduction in excess mortality with age. In the age group of 50–64, the reduction in excess mortality for women was minimal compared to the 30–49 age group, whereas for men, the reduction was substantial. In the 65+ age group, a crossover occurs between the two groups of cohabiters: divorced/widowed cohabiters then exhibited a lower mortality risk compared to their never-married cohabiters.

The control variables all behaved as expected. Being childless resulted in higher relative mortality risks for women than for men. For both men and women, the increased mortality risk for the childless decreased with age. Higher levels of education were associated with lower mortality risks. The gradient was slightly steeper for women and leveled out with age for both men and women. Placement in a higher income quantile was also associated with a lower mortality risk. There were few to no differences between the two younger age groups, whereas the gradients leveled out in the 65+ age group.

**Table 3:** Mortality risk by partnership status and age group for Swedish-born women aged 30+ in Sweden, 2012–2017

	30–49			50–64			65+		
	Model 1	Model 2	Model 3(FE)	Model 1	Model 2	Model 3(FE)	Model 1	Model 2	Model 3(FE)
Partnership status									
Married	1.00	1.00	1.00	1.00	1.00	1.00	1.00	1.00	1.00
	(.)	(.)	(.)	(.)	(.)	(.)	(.)	(.)	(.)
Never married—not cohabiting	3.20***	1.89***	1.93***	2.60***	2.14***	1.82***	1.53***	1.47***	1.87***
	(0.12)	(0.09)	(0.31)	(0.06)	(0.05)	(0.15)	(0.01)	(0.02)	(0.11)
Divorced—not cohabiting	2.31***	2.12***	1.44	1.88***	2.05***	1.88***	1.45***	1.43***	1.48***
	(0.12)	(0.12)	(0.27)	(0.04)	(0.05)	(0.14)	(0.01)	(0.01)	(0.06)
Widowed—not cohabiting	3.41***	3.32***	3.87	1.63***	1.94***	1.58**	1.21***	1.18***	1.18***
	(0.65)	(0.64)	(3.05)	(0.07)	(0.09)	(0.22)	(0.01)	(0.01)	(0.05)
Never married—cohabiting	1.12*	1.00	0.75	1.36***	1.23***	1.23*	1.45***	1.36***	1.46***
	(0.06)	(0.05)	(0.12)	(0.04)	(0.04)	(0.12)	(0.04)	(0.04)	(0.15)
Divorced/widowed—cohabiting	1.49***	1.43***	1.18	1.29***	1.41***	1.36**	1.18***	1.15***	1.16*
	(0.13)	(0.13)	(0.31)	(0.05)	(0.05)	(0.14)	(0.02)	(0.02)	(0.07)
Other	2.00***	1.72***	2.00	1.63***	1.59***	1.99*	1.67***	1.64***	1.60***
	(0.24)	(0.21)	(0.94)	(0.14)	(0.14)	(0.60)	(0.02)	(0.02)	(0.15)
Controls	No	Yes	Yes	No	Yes	Yes	No	Yes	Yes
*N*	1,274,555		1,045,262	1,090,786		869,379	1,153,737		577,762
Deaths	4,013		3,090	15,170		11,569	226,235		40,144

Controls: Childlessness, Highest level of education, Income quartile

Hazard ratio coefficients; Standard errors in parentheses; FE = Sibling Fixed Effects

For the FE model, the population is limited to individuals with differing partnership status from same-sex siblings

* $$p<0.05$$, ** $$p<0.01$$, *** $$p<0.001$$

**Table 4:** Mortality risk by partnership status and age group for Swedish-born men aged 30+ in Sweden, 2012–2017

	30–49			50–64			65+		
	Model 1	Model 2	Model 3(FE)	Model 1	Model 2	Model 3(FE)	Model 1	Model 2	Model 3(FE)
Partnership status									
Married	1.00	1.00	1.00	1.00	1.00	1.00	1.00	1.00	1.00
	(.)	(.)	(.)	(.)	(.)	(.)	(.)	(.)	(.)
Never married—not cohabiting	4.79***	3.18***	2.90***	3.66***	2.27***	2.26***	1.85***	1.62***	2.01***
	(0.15)	(0.12)	(0.37)	(0.06)	(0.04)	(0.15)	(0.01)	(0.02)	(0.10)
Divorced—not cohabiting	3.66***	3.24***	2.99***	2.89***	2.36***	2.07***	1.69***	1.60***	1.80***
	(0.17)	(0.16)	(0.53)	(0.06)	(0.05)	(0.13)	(0.01)	(0.01)	(0.07)
Widowed—not cohabiting	2.67**	2.54**	1.38	1.94***	1.79***	1.86**	1.19***	1.16***	1.30***
	(0.81)	(0.77)	(1.36)	(0.12)	(0.11)	(0.36)	(0.01)	(0.01)	(0.06)
Never married—cohabiting	1.16***	1.05	1.13	1.29***	1.11***	1.21*	1.28***	1.16***	1.19*
	(0.05)	(0.05)	(0.15)	(0.04)	(0.03)	(0.10)	(0.02)	(0.02)	(0.08)
Divorced/widowed—cohabiting	1.69***	1.64***	2.65***	1.22***	1.15***	1.20	1.11***	1.07***	1.02
	(0.14)	(0.14)	(0.70)	(0.04)	(0.04)	(0.12)	(0.01)	(0.01)	(0.05)
Other	2.04***	1.74***	0.81	2.26***	1.85***	1.90**	1.66***	1.63***	1.70***
	(0.23)	(0.20)	(0.29)	(0.14)	(0.12)	(0.41)	(0.02)	(0.02)	(0.15)
Controls	No	Yes	Yes	No	Yes	Yes	No	Yes	Yes
*N*	1,343,716		1,102,904	1,120,978		897,241	1,009,163		565,872
Deaths	6,924		5,205	22,719		17,363	200,302		55,354

Controls: Childlessness, Highest level of education, Income quartile

Hazard ratio coefficients; Standard errors in parentheses; FE = Sibling Fixed Effects

Note: For the FE model, the population is limited to individuals with differing partnership status from same-sex siblings

* $$p<0.05$$, ** $$p<0.01$$, *** $$p<0.001$$

Model 3 is the sibling fixed effects model, which had the same variables as Model 2 but only compared siblings with different partnership statuses when at least one sibling died during the study period. Thus, the model accounted for social background factors that were shared between siblings (parental education, income, neighborhoods during upbringing, etc.). The results did not differ to a substantial extent and largely confirmed our main findings. The sibling models had larger standard errors, and effects were in more cases not statistically significant. Typically, the effect sizes were smaller in the sibling effects models. This may be due to the fact that these models capture more dimensions of SES (both in the family of origin, and for the individual themselves) than only observable individual-level controls.

In addition to the three models, we ran separate regressions with each control (Appendix 1) to assess the amount of variation explained by each of the partnership statuses. Childlessness explained almost none of the excess mortality for the divorced and widowed, both cohabiting and not, indicating that the increased mortality risk for divorced/widowed individuals is related to their low SES. For those who never married, both cohabiting and not, childlessness explained some of the excess mortality, especially for women, for whom it was a stronger predictor than education and income. This indicates that some of the excess mortality for the never-married individuals is due to them being, to a larger extent than those of other partnership statuses, childless and low SES. Further, we ran a model with marriage status instead of partnership status as the independent variable to allow for comparisons of our results to studies where marriage status is the focus. In this model, the mortality differences between the married the other statuses are smaller due to how non-marital cohabiters are being categorized as single, divorced or widowed (Appendix 1).

## Discussion

In this study, we used Swedish register data to analyze how partnership status is associated with mortality and how the association varies across life stages. Previous literature has primarily focused on marriage status, and few studies have included cohabitation. Those that do have found that mortality varies within the cohabiting group. This variation may be due to the different meanings and implications of cohabitation at different life stages. Cohabitation may serve distinct social, economic, and emotional functions at young, middle, and older ages, leading to diverse health outcomes across these age groups. The contribution and strength of the present study is the rich longitudinal data of the total population that allows us to include marriage histories to distinguish between cohabitation among the never married and cohabitation among the previously married. In addition, we included information on family structures to employ sibling comparisons, which allowed us to examine whether disadvantages from certain partnership statuses can exist net of family-of-origin background factors. In addition, this is the first mortality study to account for cohabitation in the Swedish context.

Our results confirm that married individuals in Sweden experience the lowest mortality rate at all ages. The degree of mortality advantage compared to cohabiters varies by age. For the youngest age group, there is no significant difference between the married and cohabiters who have never been married, whereas at later ages, excess mortality among the cohabiting is more pronounced. However, cohabitation is associated with reduced mortality at all ages compared to those who are unpartnered, regardless of whether cohabitation is observed among the never married or the previously married. After using sibling fixed effects, our main covariates were largely unchanged. This suggests that higher mortality among single individuals and lower mortality among married individuals is not due to confounding by social disadvantage in the family of origin, though social disadvantage not shared with siblings (e.g., events during the individual’s own life course, such as mental health issues or substance abuse) cannot be controlled for in this way. To account for such factors requires individual-level fixed effects, which require repeatable outcome measures (Hu et al., 2019; van Hedel et al., 2018) not applicable in this case.

Our most significant finding is that the patterns of excess mortality among cohabiters differ distinctly between those who have never been married and those who cohabit after marriage. This was first suggested by sample data from England and Wales (Franke & Kulu, 2018). Our study finds strong support for a mortality crossover across age groups for these two groups of cohabiters. At younger ages never-married cohabiters are indistinguishable from their married counterparts in terms of mortality risk, while at older ages they have an elevated mortality risk. For divorced/widowed cohabiters, the trend is reversed: relative mortality is substantially higher at younger ages and decreases with age.

To interpret these findings, we should first consider how selection mechanisms into and out of certain partnership statuses change across the life course. Cohabitation before marriage is the norm in Sweden. However, cohabitation has not replaced marriage, and most long-term cohabiters eventually marry (Sobotka & Toulemon, 2008; Ohlsson-Wijk et al., 2020). For example, over 50% of the Swedish population experiences marriage by age 37 and almost 70% by age 50 (Andersson et al., 2017). Thus, cohabitation and subsequent marriage represent the most normative life course trajectory in early adulthood in Sweden, with many young cohabiting couples expected to marry. Based on our results, the selection processes that influence couples to remain in cohabitation or to transition to marriage do not seem to differ significantly enough to produce mortality differences between cohabiters and the married in younger ages.

The higher mortality risk among never-married cohabiters at older ages could partly be due to either differences in the long-term protective effects between marriage and cohabitation or to the group including serial cohabiters and those who have been single for most of their lives, two groups who have been shown to experience high mortality (Sassler & Lichter, 2020). This group might also suffer from increased selectivity in smaller groups. Hu Goldman (1990) made the observation that mortality risks were inversely related to the size of the marriage status group. In contrast to selection, stress and isolation may also be important if stigma and discrimination are more common in a smaller and rarer group. Our findings are largely consistent with such arguments, as we consistently observe higher mortality for civil status groups that are rarer in the population.

In turn, young divorced or widowed cohabiters consist of a small group who get divorced or widowed at an early age and later repartner into a cohabiting union. Their mortality is between those not in a union and those in a union with no past marriages. Likely, there is health selection among the divorced and widowed population in that the healthiest are more likely to repartner (Hu Goldman, 1990). Further, in terms of causal effects, a new partner might lessen the effects of bereavement (Bowling, 1987) or the increased stress following a dissolved marriage (Hemström, 1996). In older age groups, the decreasing excess mortality for divorced and widowed cohabiters could be attributed to there being a larger share who have gained the protective effects from longer-lasting marriages before union dissolution.

Our results do not directly support the notion of accumulation of advantage for the married compared to cohabiters. These hypothesized effects primarily concern health and wealth benefits that accumulates over time, potentially due to greater union satisfaction, higher expected stability and commitment, or an increased tendency to pool economic resources within marriage (Carr & Springer, 2010). In our data, the advantage for the married decreases for each older age group. However, this is not the focus of the present paper, and to test this one would need to include union length and timing of trajectories. This is a possible avenue for further research in this area.

While it is reasonable to interpret our results as reflective of differences by age and life stage, one should be mindful that they have been measured for distinct cohorts and may partly reflect cohort-specific experiences for those individuals. As the time window of this study was only six years, we could not separately identify age and cohort effects. Studying how age patterns in union formation and mortality have changed across cohorts is an interesting topic for future research, given the 20th century reversals in SES gradients in cohabitation (Perelli-Harris et al., 2010) and evidence of increasingly positive SES gradients for both longevity and entry into marriage and partnership.

The present study confirms results from previous literature on the association between marriage status and mortality, such as mortality differences decreasing with age (Staehelin et al., 2012; Franke & Kulu, 2018; Koskinen et al., 2007). We further found that the increased mortality risk from being single was greater for men than for women and that the difference between being married and cohabiting was larger for women in the two older age groups. These types of gender differences have been theorized to be due to men having an increased tendency toward unhealthy behaviors and benefiting more from the social support from their spouse (Umberson, 1987), whereas women benefit more than men from the economic advantages of marriage (Carlsson et al., 2014; Staehelin et al., 2012). One interpretation is that the latter is not as present in cohabiting unions as in marriages, whereas the two former mechanisms are, resulting in men in cohabiting unions enjoying the advantage of social control and support, while women miss out on the economic advantages.

The present study is unable to determine to what degree the findings represent patterns that will generalize to other countries. Sweden has gained recognition as a forerunner (Sobotka & Toulemon, 2008) in family-related advancements, showcasing early trends in union formation. However, it remains uncertain whether this holds true for mortality rates among individuals in cohabiting unions, thereby prompting the need for future investigations.

One limitation of this study is the validity of the dwelling register used to determine cohabitation status. Given its heuristic nature, which derives cohabitation as a partnership status, some degree of misclassification is inevitable. For example, in households where there are more than two potential cohabiters, none can be classified. Similarly, individuals cohabiting with a new partner while still formally married cannot be properly classified. This likely results in an underestimation of cohabiting unions by approximately 10% (Statistics, 2017). Furthermore, individuals might cohabit while their recorded residences are at separate dwellings, which leads to a risk of overestimating the population living alone. At older ages, there are additional reasons why two individuals in a romantic union might abstain from cohabitation. For example, two established households might prefer “living apart together” (Olah et al., 2020) due to previous family conditions or children from previous partners. The different-sex definition of cohabitation also did not allow for estimation of cohabitation patterns for same-sex partners. Additional limitations include the absence of data on health or health behavior, which would have allowed us to distinguish selection mechanisms from causal mechanisms more effectively. Furthermore, the study’s relatively short follow-up period, combined with the left-truncation of the data, limited the number of observed deaths, especially among younger participants. These factors also restricted our ability to use detailed partnership histories for our comparisons. For instance, we were unable to distinguish between first marriages and remarriages due to this data limitations. However, to gain a more comprehensive understanding of the effects of cohabitation vs marriage, it is also necessary to compare cohabiters who were previously married with those who are married but were previously divorced. Unfortunately, we must leave this interesting comparison to future research.

This study explored the association between partnership status and mortality, focusing on the differences between marriage and cohabitation. A significant contribution to the current knowledge is the differentiation between cohabiters who have never been married and those who cohabit after marriage. Young, never-married cohabiters demonstrate mortality risks similar to those of their married counterparts, while at older ages, they face an elevated mortality risk. On the other hand, divorced or widowed cohabiters experience higher mortality risks at younger ages, which gradually decrease with age. These findings underscore that cohabitation must be treated as a diverse partnership status in future research, as cohabitation is a reflection of different selection patterns and life trajectories at different ages. With cohabitation becoming more common, not least among older individuals for each subsequent cohort, this will only become more important in the coming decades. Future demographers should think carefully about how to interpret differences between partnership statuses, as cohabitation implies different things at different life stages.

## Figures and Tables

**Fig. 2:**
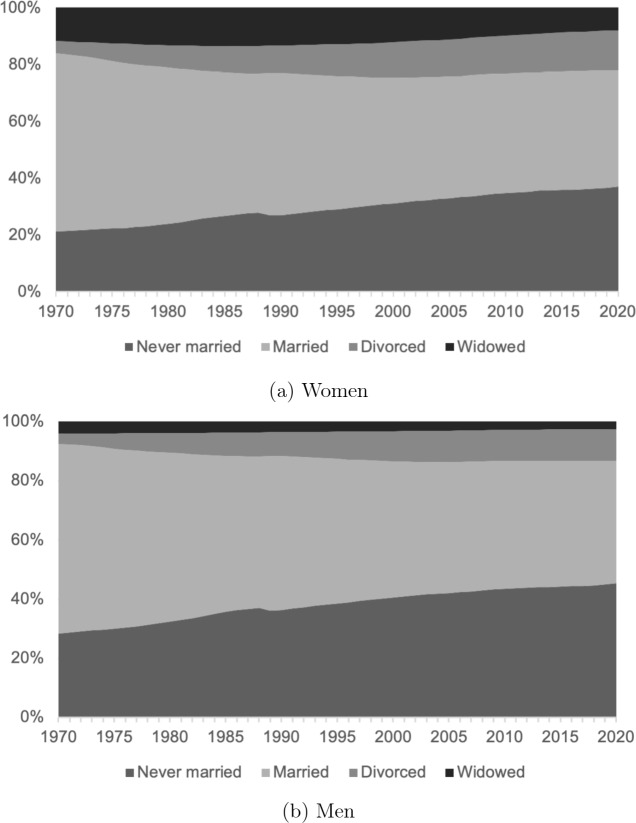
Distribution of marriage status for Swedish population aged 18+ between 1970 and 2020 by sex. Source: Statistics Sweden

## Data Availability

The register data that support the findings of this study are held by Statistics Sweden but restrictions apply to the availability of these data, which were used under license for the current study, and so are not publicly available nor available for us to share unless the research has a relevant ethical approval.

## Appendix

### Marriage Status in Sweden 1970–2020

See Fig. 2.

### Full Regression Tables

See Tables 5 and 6.

**Table 5:** Mortality risk by partnership status and age group for Swedish-born women aged 30+ in Sweden, 2012–2017

	30–49			50–64			65+		
	1	2	3(FE)	1	2	3(FE)	1	2	3(FE)
Partnership status									
Married (ref.)	1.00	1.00	1.00	1.00	1.00	1.00	1.00	1.00	1.00
	(.)	(.)	(.)	(.)	(.)	(.)	(.)	(.)	(.)
Never married—not cohabiting	3.20***	1.89***	1.93***	2.60***	2.14***	1.82***	1.53***	1.47***	1.87***
	(0.12)	(0.09)	(0.31)	(0.06)	(0.05)	(0.15)	(0.01)	(0.02)	(0.11)
Divorced—not cohabiting	2.31***	2.12***	1.44	1.88***	2.05***	1.88***	1.45***	1.43***	1.48***
	(0.12)	(0.12)	(0.27)	(0.04)	(0.05)	(0.14)	(0.01)	(0.01)	(0.06)
Widowed—not cohabiting	3.41***	3.32***	3.87	1.63***	1.94***	1.58**	1.21***	1.18***	1.18***
	(0.65)	(0.64)	(3.05)	(0.07)	(0.09)	(0.22)	(0.01)	(0.01)	(0.05)
Never married—cohabiting	1.12*	1.00	0.75	1.36***	1.23***	1.23*	1.45***	1.36***	1.46***
	(0.06)	(0.05)	(0.12)	(0.04)	(0.04)	(0.12)	(0.04)	(0.04)	(0.15)
Divorced/widowed—cohabiting	1.49***	1.43***	1.18	1.29***	1.41***	1.36**	1.18***	1.15***	1.16*
	(0.13)	(0.13)	(0.31)	(0.05)	(0.05)	(0.14)	(0.02)	(0.02)	(0.07)
Other	2.00***	1.72***	2.00	1.63***	1.59***	1.99*	1.67***	1.64***	1.60***
	(0.24)	(0.21)	(0.94)	(0.14)	(0.14)	(0.60)	(0.02)	(0.02)	(0.15)
Childless									
No (ref.)		1.00	1.00		1.00	1.00		1.00	1.00
		(.)	(.)		(.)	(.)		(.)	(.)
Yes		2.55***	2.49***		1.53***	1.66***		1.09***	1.28***
		(0.10)	(0.38)		(0.03)	(0.13)		(0.01)	(0.06)
Highest level of education									
Elementary or lower		2.05***	1.54*		1.33***	1.27***		1.10***	1.10**
		(0.09)	(0.28)		(0.03)	(0.09)		(0.01)	(0.04)
Upper secondary (ref.)		1.00	1.00		1.00	1.00		1.00	1.00
		(.)	(.)		(.)	(.)		(.)	(.)
Higher education less than 2 years		0.88	0.62*		0.82***	0.89		0.89***	0.91
		(0.06)	(0.14)		(0.04)	(0.13)		(0.02)	(0.11)
Higher education at least 2 years		0.65***	0.56***		0.72***	0.81**		0.81***	0.76***
		(0.03)	(0.09)		(0.02)	(0.06)		(0.01)	(0.04)
Doctoral education		0.47**	0.00		0.49***	0.35*		0.71***	0.55*
		(0.11)	(0.00)		(0.07)	(0.16)		(0.04)	(0.14)
Missing		2.98***	5.97		2.45***	2.85*		0.99	1.66
		(0.34)	(6.32)		(0.21)	(1.35)		(0.02)	(0.44)
Income quartile									
0–25		1.34***	1.06		1.82***	1.51***		0.95***	1.06
		(0.05)	(0.14)		(0.04)	(0.09)		(0.01)	(0.04)
26–50 (ref.)		1.00	1.00		1.00	1.00		1.00	1.00
		(.)	(.)		(.)	(.)		(.)	(.)
51–75		0.61***	0.83		0.70***	0.70***		0.87***	0.82***
		(0.03)	(0.14)		(0.02)	(0.05)		(0.01)	(0.03)
76–100		0.52***	0.71		0.74***	0.67***		0.87***	0.79***
		(0.03)	(0.13)		(0.02)	(0.06)		(0.01)	(0.04)
*N*	1,274,555		1,045,262	1,090,786		869,379	1,153,737		577,762
Deaths	4,013		3,090	15,170		11,569	226,235		40,144

Hazard ratio coefficients; Standard errors in parentheses; FE = Sibling Fixed Effects

For the FE model, the population is limited to individuals with differing partnership status from same-sex siblings

* $$p<0.05$$, ** $$p<0.01$$, *** $$p<0.001$$

**Table 6:** Mortality risk by partnership status and age group for Swedish-born men aged 30+ in Sweden, 2012–2017

	30–49			50–64			65+		
	1	2	3(FE)	1	2	3(FE)	1	2	3(FE)
Partnership status									
Married (ref.)	1.00	1.00	1.00	1.00	1.00	1.00	1.00	1.00	1.00
	(.)	(.)	(.)	(.)	(.)	(.)	(.)	(.)	(.)
Never married—not cohabiting	4.79***	3.18***	2.90***	3.66***	2.27***	2.26***	1.85***	1.62***	2.01***
	(0.15)	(0.12)	(0.37)	(0.06)	(0.04)	(0.15)	(0.01)	(0.02)	(0.10)
Divorced—not cohabiting	3.66***	3.24***	2.99***	2.89***	2.36***	2.07***	1.69***	1.60***	1.80***
	(0.17)	(0.16)	(0.53)	(0.06)	(0.05)	(0.13)	(0.01)	(0.01)	(0.07)
Widowed—not cohabiting	2.67**	2.54**	1.38	1.94***	1.79***	1.86**	1.19***	1.16***	1.30***
	(0.81)	(0.77)	(1.36)	(0.12)	(0.11)	(0.36)	(0.01)	(0.01)	(0.06)
Never married—cohabiting	1.16***	1.05	1.13	1.29***	1.11***	1.21*	1.28***	1.16***	1.19*
	(0.05)	(0.05)	(0.15)	(0.04)	(0.03)	(0.10)	(0.02)	(0.02)	(0.08)
Divorced/widowed—cohabiting	1.69***	1.64***	2.65***	1.22***	1.15***	1.20	1.11***	1.07***	1.02
	(0.14)	(0.14)	(0.70)	(0.04)	(0.04)	(0.12)	(0.01)	(0.01)	(0.05)
Other	2.04***	1.74***	0.81	2.26***	1.85***	1.90**	1.66***	1.63***	1.70***
	(0.23)	(0.20)	(0.29)	(0.14)	(0.12)	(0.41)	(0.02)	(0.02)	(0.15)
Childless									
No (ref.)		1.00	1.00		1.00	1.00		1.00	1.00
		(.)	(.)		(.)	(.)		(.)	(.)
Yes		1.40***	1.49***		1.18***	1.29***		1.04***	1.07
		(0.04)	(0.16)		(0.02)	(0.08)		(0.01)	(0.04)
Highest level of education									
Elementary or lower		1.69***	1.15		1.15***	1.11		1.05***	1.07*
		(0.05)	(0.14)		(0.02)	(0.06)		(0.01)	(0.03)
Upper secondary (ref.)		1.00	1.00		1.00	1.00		1.00	1.00
		(.)	(.)		(.)	(.)		(.)	(.)
Higher education less than 2 years		0.75***	0.82		0.80***	0.82		0.93***	0.95
		(0.04)	(0.16)		(0.02)	(0.08)		(0.02)	(0.07)
Higher education at least 2 years		0.55***	0.67**		0.71***	0.86		0.87***	0.93
		(0.02)	(0.10)		(0.02)	(0.07)		(0.01)	(0.04)
Doctoral education		0.69*	0.66		0.58***	0.97		0.76***	0.77*
		(0.10)	(0.32)		(0.05)	(0.26)		(0.02)	(0.10)
Missing		1.78***	2.14		1.94***	1.08		1.06*	1.14
		(0.17)	(1.03)		(0.13)	(0.28)		(0.03)	(0.22)
Income quartile									
0–25		1.44***	1.43**		1.78***	1.58***		0.95***	1.06
		(0.04)	(0.17)		(0.03)	(0.10)		(0.01)	(0.04)
26–50 (ref.)		1.00	1.00		1.00	1.00		1.00	1.00
		(.)	(.)		(.)	(.)		(.)	(.)
51–75		0.55***	0.69**		0.70***	0.69***		0.87***	0.84***
		(0.02)	(0.08)		(0.01)	(0.04)		(0.01)	(0.03)
76–100		0.42***	0.53***		0.62***	0.69***		0.78***	0.72***
		(0.02)	(0.07)		(0.01)	(0.05)		(0.01)	(0.03)
*N*	1,343,716		1,102,904	1,120,978		897,241	1,009,163		565,872
Deaths	6,924		5,205	22,719		17,363	200,302		55,354

Hazard ratio coefficients; Standard errors in parentheses; FE = Sibling Fixed Effects

For the FE model, the population is limited to individuals with differing partnership status from same-sex siblings

* $$p<0.05$$, ** $$p<0.01$$, *** $$p<0.001$$

### Marriage Status Regressions

See Table 7.

**Table 7:** Mortality risk by marriage status and age group for Swedish-born population aged 30+ in Sweden, 2012–2017

	Men			Women		
	30–49	50–64	65+	30–49	50–64	65+
Marriage status						
Married (ref.)	1.00	1.00	1.00	1.00	1.00	1.00
	(.)	(.)	(.)	(.)	(.)	(.)
Never married	3.04***	2.79***	1.74***	2.06***	2.11***	1.52***
	(0.09)	(0.04)	(0.01)	(0.07)	(0.04)	(0.01)
Divorced	2.97***	2.35***	1.53***	2.03***	1.72***	1.41***
	(0.13)	(0.04)	(0.01)	(0.10)	(0.04)	(0.01)
Widowed	2.16**	1.72***	1.18***	3.06***	1.57***	1.20***
	(0.63)	(0.10)	(0.01)	(0.54)	(0.07)	(0.01)
Other	2.04***	2.26***	1.66***	2.00***	1.64***	1.66***
	(0.23)	(0.14)	(0.02)	(0.24)	(0.14)	(0.02)
*N*	1,343,716	1,120,978	1,009,163	1,274,555	1,090,786	1,153,737
Deaths	6,924	22,719	200,302	4,013	15,170	226,235

Hazard ratio coefficients; Standard errors in parentheses

* $$p<0.05$$, ** $$p<0.01$$, *** $$p<0.001$$

### Regressions with Gender Interaction

See Table 8.

**Table 8:** Mortality risk by partnership status and age group for Swedish-born population aged 30+ in Sweden, 2012–2017 - Interaction term for sex and partnership status

	30–49		50-64		65+	
	1	2	1	2	1	2
Partnership status						
Married (ref.)	1.00	1.00	1.00	1.00	1.00	1.00
Never married—not cohabiting	4.85***	2.75***	3.65***	2.14***	1.84***	1.59***
	(0.15)	(0.10)	(0.06)	(0.04)	(0.01)	(0.01)
Divorced—not cohabiting	3.61***	3.14***	2.89***	2.37***	1.68***	1.61***
	(0.17)	(0.15)	(0.06)	(0.05)	(0.01)	(0.01)
Widowed—not cohabiting	2.63**	2.36**	1.95***	1.78***	1.16***	1.13***
	(0.79)	(0.72)	(0.12)	(0.11)	(0.01)	(0.01)
Never married—cohabiting	1.18***	1.04	1.29***	1.08**	1.28***	1.14***
	(0.05)	(0.05)	(0.04)	(0.03)	(0.02)	(0.02)
Divorced/widowed—cohabiting	1.68***	1.61***	1.22***	1.16***	1.11***	1.07***
	(0.14)	(0.14)	(0.04)	(0.04)	(0.01)	(0.01)
Other	2.06***	1.70***	2.25***	1.83***	1.64***	1.62***
	(0.23)	(0.19)	(0.14)	(0.12)	(0.02)	(0.02)
Sex						
Male (ref.)	1.00	1.00	1.00	1.00	1.00	1.00
Female	0.85***	0.73***	0.84***	0.65***	0.69***	0.65***
	(0.03)	(0.03)	(0.01)	(0.01)	(0.00)	(0.00)
Interaction						
Married*Female (ref.)	1.00	1.00	1.00	1.00	1.00	1.00
NeverM-NoCohab*Female	0.65***	0.85***	0.72***	1.09**	0.85***	0.94***
	(0.03)	(0.04)	(0.02)	(0.03)	(0.01)	(0.01)
Divored-NoCohab*Female	0.65***	0.72***	0.65***	0.87***	0.87***	0.89***
	(0.05)	(0.05)	(0.02)	(0.03)	(0.01)	(0.01)
Widowed-NoCohab*Female	1.34	1.57	0.83*	1.10	1.06***	1.06***
	(0.48)	(0.56)	(0.06)	(0.08)	(0.01)	(0.01)
NeverM-Cohab*Female	0.93	0.97	1.06	1.18***	1.14***	1.21***
	(0.06)	(0.06)	(0.04)	(0.05)	(0.04)	(0.04)
Div/wid-Cohab*Female	0.91	0.92	1.06	1.22***	1.06**	1.07***
	(0.11)	(0.11)	(0.05)	(0.06)	(0.02)	(0.02)
Other*Female	0.96	1.03	0.73**	0.89	1.02	1.02
	(0.16)	(0.17)	(0.08)	(0.10)	(0.02)	(0.02)
Childless						
No (ref.)		1.00		1.00		1.00
Yes		1.77***		1.30***		1.07***
		(0.04)		(0.02)		(0.00)
Highest level of education						
Elementary or lower		1.80***		1.22***		1.08***
		(0.05)		(0.02)		(0.00)
Upper secondary (ref.)		1.00		1.00		1.00
		(.)		(.)		(.)
Higher education less than 2 years		0.79***		0.81***		0.92***
		(0.03)		(0.02)		(0.01)
Higher education at least 2 years		0.59***		0.71***		0.85***
		(0.02)		(0.01)		(0.00)
Doctoral education		0.61***		0.56***		0.75***
		(0.08)		(0.04)		(0.02)
Missing		2.18***		2.12***		1.02
		(0.16)		(0.11)		(0.02)
Income quartile						
0–25		1.39***		1.80***		0.95***
		(0.03)		(0.02)		(0.00)
26–50 (ref.)		1.00		1.00		1.00
		(.)		(.)		(.)
51–75		0.58***		0.71***		0.88***
		(0.02)		(0.01)		(0.00)
76–100		0.45***		0.66***		0.82***
		(0.01)		(0.01)		(0.00)
*N*	2,618,271	2,618,271	2,211,764	2,211,764	2,162,900	2,162,900
Deaths	10,937	10,937	37,889	37,889	426,537	426,537

Hazard ratio coefficients; Standard errors in parentheses

* $$p<0.05$$, ** $$p<0.01$$, *** $$p<0.001$$

### Regressions with Fixed Effects Population

See Table 9.

**Table 9:** Mortality risk by partnership status and age group for Swedish-born population (30+) with differing partnership status from same-sex siblings, 2012–2017

	Men			Women		
	30–49	50–64	65+	30–49	50–64	65+
Partnership status						
Married (ref.)	1.00	1.00	1.00	1.00	1.00	1.00
	(.)	(.)	(.)	(.)	(.)	(.)
Never married—not cohabiting	3.24***	1.90***	2.26***	2.19***	1.96***	1.79***
	(0.14)	(0.10)	(0.05)	(0.06)	(0.03)	(0.04)
Divorced—not cohabiting	3.18***	2.15***	2.34***	2.06***	1.88***	1.65***
	(0.18)	(0.13)	(0.05)	(0.05)	(0.02)	(0.02)
Widowed—not cohabiting	3.01***	3.37***	1.79***	1.91***	1.35***	1.29***
	(0.96)	(0.73)	(0.12)	(0.10)	(0.02)	(0.02)
Never married—cohabiting	1.07	0.97	1.10**	1.21***	1.17***	1.37***
	(0.05)	(0.06)	(0.03)	(0.04)	(0.03)	(0.05)
Divorced/widowed—cohabiting	1.70***	1.43***	1.17***	1.39***	1.07***	1.13***
	(0.16)	(0.14)	(0.05)	(0.06)	(0.02)	(0.03)
Other	1.55**	1.67***	1.68***	1.66***	1.83***	1.80***
	(0.21)	(0.24)	(0.13)	(0.16)	(0.05)	(0.06)
Childless						
No (ref.)	1.00	1.00	1.00	1.00	1.00	1.00
	(.)	(.)	(.)	(.)	(.)	(.)
Yes	1.36***	2.68***	1.18***	1.54***	1.05***	1.20***
	(0.05)	(0.12)	(0.02)	(0.04)	(0.01)	(0.02)
Highest level of education						
Elementary or lower	1.66***	1.93***	1.17***	1.34***	1.08***	1.18***
	(0.06)	(0.10)	(0.02)	(0.03)	(0.01)	(0.01)
Upper secondary (ref.)	1.00	1.00	1.00	1.00	1.00	1.00
	(.)	(.)	(.)	(.)	(.)	(.)
Higher education less than 2 years	0.75***	0.92	0.79***	0.83***	0.93*	0.88**
	(0.04)	(0.07)	(0.03)	(0.05)	(0.03)	(0.04)
Higher education at least 2 years	0.58***	0.66***	0.73***	0.73***	0.82***	0.73***
	(0.02)	(0.03)	(0.02)	(0.02)	(0.01)	(0.01)
Doctoral education	0.75	0.45**	0.64***	0.57***	0.72***	0.69***
	(0.12)	(0.12)	(0.06)	(0.09)	(0.03)	(0.06)
Missing	2.13***	3.32***	1.97***	2.58***	1.39***	1.47***
	(0.23)	(0.43)	(0.15)	(0.25)	(0.08)	(0.11)
Income quartile						
0–25	1.48***	1.30***	1.77***	1.81***	1.05***	1.01
	(0.05)	(0.06)	(0.04)	(0.04)	(0.01)	(0.01)
26–50 (ref.)	1.00	1.00	1.00	1.00	1.00	1.00
	(.)	(.)	(.)	(.)	(.)	(.)
51–75	0.58***	0.59***	0.71***	0.69***	0.80***	0.81***
	(0.02)	(0.03)	(0.02)	(0.02)	(0.01)	(0.01)
76–100	0.44***	0.51***	0.63***	0.71***	0.67***	0.77***
	(0.02)	(0.03)	(0.02)	(0.03)	(0.01)	(0.01)
Missing	0.00	0.00	0.00	0.00	0.00	0.00
	(.)	(.)	(.)	(0.00)	(.)	(.)
*N*	1,102,904	1,045,262	897,241	869,379	565,872	577,762
Deaths	5,205	3,090	17,363	11,569	55,354	40,144

Hazard ratio coefficients; Standard errors in parentheses

* $$p<0.05$$, ** $$p<0.01$$, *** $$p<0.001$$

### Regressions with Separate Addition of Control Variables

See Table 10, 11, 12, 13, 14 and 15.

**Table 10:** Mortality risk by partnership status for Swedish-born men aged 30–49 in Sweden, 2012–2017

	1	1a	1b	2
Partnership status				
Married (ref.)	1.00	1.00	1.00	1.00
	(.)	(.)	(.)	(.)
Never married—not cohabiting	4.79***	4.08***	3.91***	3.18***
	(0.15)	(0.15)	(0.13)	(0.12)
Divorced—not cohabiting	3.66***	3.60***	3.32***	3.24***
	(0.17)	(0.17)	(0.16)	(0.16)
Widowed—not cohabiting	2.67**	2.58**	2.69**	2.54**
	(0.81)	(0.78)	(0.81)	(0.77)
Never married—cohabiting	1.16***	1.12**	1.09	1.05
	(0.05)	(0.05)	(0.05)	(0.05)
Divorced/widowed—cohabiting	1.69***	1.68***	1.65***	1.64***
	(0.14)	(0.14)	(0.14)	(0.14)
Other	2.04***	1.99***	1.81***	1.74***
	(0.23)	(0.22)	(0.20)	(0.20)
Childless				
No (ref.)		1.00		1.00
		(.)		(.)
Yes		1.29***		1.40***
		(0.04)		(0.04)
Highest level of education				
Elementary or lower			1.69***	1.69***
			(0.05)	(0.05)
Upper secondary (ref.)			1.00	1.00
			(.)	(.)
Higher education less than 2 years			0.77***	0.75***
			(0.04)	(0.04)
Higher education at least 2 years			0.56***	0.55***
			(0.02)	(0.02)
Doctoral education			0.70**	0.69*
			(0.10)	(0.10)
Missing			1.94***	1.78***
			(0.18)	(0.17)
Income quartile				
0–25			1.47***	1.44***
			(0.05)	(0.04)
26–50 (ref.)			1.00	1.00
			(.)	(.)
51–75			0.56***	0.55***
			(0.02)	(0.02)
76–100			0.43***	0.42***
			(0.02)	(0.02)
Missing			0.00	0.00
			(.)	(.)
*N*	1,343,716	1,343,716	1,343,716	1,343,716
Deaths	6,924	6,924	6,924	6,924

Hazard ratio coefficients; Standard errors in parentheses

* $$p<0.05$$, ** $$p<0.01$$, *** $$p<0.001$$

**Table 11:** Mortality risk by partnership status for Swedish-born men aged 50–64 in Sweden, 2012–2017

	1	1a	1b	2
Partnership status				
Married (ref.)	1.00	1.00	1.00	1.00
	(.)	(.)	(.)	(.)
Never married—not cohabiting	3.66***	3.12***	2.49***	2.27***
	(0.06)	(0.06)	(0.04)	(0.04)
Divorced—not cohabiting	2.89***	2.88***	2.36***	2.36***
	(0.06)	(0.06)	(0.05)	(0.05)
Widowed—not cohabiting	1.94***	1.89***	1.81***	1.79***
	(0.12)	(0.11)	(0.11)	(0.11)
Never married—cohabiting	1.29***	1.23***	1.14***	1.11***
	(0.04)	(0.03)	(0.03)	(0.03)
Divorced/widowed—cohabiting	1.22***	1.23***	1.15***	1.15***
	(0.04)	(0.04)	(0.04)	(0.04)
Other	2.26***	2.20***	1.87***	1.85***
	(0.14)	(0.14)	(0.12)	(0.12)
Childless				
No (ref.)		1.00		1.00
		(.)		(.)
Yes		1.31***		1.18***
		(0.02)		(0.02)
Highest level of education				
Elementary or lower			1.16	1.15***
			(0.02)	(0.02)
Upper secondary (ref.)			1.00	1.00
			(.)	(.)
Higher education less than 2 years			0.81***	0.80***
			(0.02)	(0.02)
Higher education at least 2 years			0.71***	0.71***
			(0.02)	(0.02)
Doctoral education			0.59***	0.58***
			(0.05)	(0.05)
Missing			2.02***	1.94***
			(0.13)	(0.13)
Income quartile				
0–25			1.80***	1.78***
			(0.03)	(0.03)
26–50 (ref.)			1.00	1.00
			(.)	(.)
51–75			0.70***	0.70***
			(0.01)	(0.01)
76–100			0.62***	0.62***
			(0.01)	(0.01)
Missing			0.00	0.00
			(.)	(.)
*N*	1,120,978	1,120,978	1,120,978	1,120,978
Deaths	22,719	22,719	22,719	22,719

Hazard ratio coefficients; Standard errors in parentheses

* $$p<0.05$$, ** $$p<0.01$$, *** $$p<0.001$$

**Table 12:** Mortality risk by partnership status for Swedish-born men aged 65 and above in Sweden, 2012–2017

	1	1a	1b	2
Partnership status				
Married (ref.)	1.00	1.00	1.00	1.00
	(.)	(.)	(.)	(.)
Never married—not cohabiting	1.85***	1.77***	1.67***	1.62***
	(0.01)	(0.02)	(0.01)	(0.02)
Divorced—not cohabiting	1.69***	1.68***	1.60***	1.60***
	(0.01)	(0.01)	(0.01)	(0.01)
Widowed—not cohabiting	1.19***	1.19***	1.16***	1.16***
	(0.01)	(0.01)	(0.01)	(0.01)
Never married—cohabiting	1.28***	1.24***	1.18***	1.16***
	(0.02)	(0.02)	(0.02)	(0.02)
Divorced/widowed—cohabiting	1.11***	1.11***	1.07***	1.07***
	(0.01)	(0.01)	(0.01)	(0.01)
Other	1.66***	1.66***	1.63***	1.63***
	(0.02)	(0.02)	(0.02)	(0.02)
Childless				
No (ref.)		1.00		1.00
		(.)		(.)
Yes		1.05***		1.04***
		(0.01)		(0.01)
Highest level of education				
Elementary or lower			1.05***	1.05***
			(0.01)	(0.01)
Upper secondary (ref.)			1.00	1.00
			(.)	(.)
Higher education less than 2 years			0.93***	0.93***
			(0.02)	(0.02)
Higher education at least 2 years			0.87***	0.87***
			(0.01)	(0.01)
Doctoral education			0.76***	0.76***
			(0.02)	(0.02)
Missing			1.06**	1.06*
			(0.03)	(0.03)
Income quartile				
0–25			0.96***	0.95***
			(0.01)	(0.01)
26–50 (ref.)			1.00	1.00
			(.)	(.)
51–75			0.87***	0.87***
			(0.01)	(0.01)
76–100			0.78***	0.78***
			(0.01)	(0.01)
Missing			0.00	0.00
			(0.00)	(0.00)
*N*	1,009,163	1,009,163	1,009,163	1,009,163
Deaths	200,302	200,302	200,302	200,302

Hazard ratio coefficients; Standard errors in parentheses

* $$p<0.05$$, ** $$p<0.01$$, *** $$p<0.001$$

**Table 13:** Mortality risk by partnership status for Swedish-born women aged 30–49 in Sweden, 2012–2017

	1	1a	1b	2
Partnership status				
Married (ref.)	1.00	1.00	1.00	1.00
	(.)	(.)	(.)	(.)
Never married—not cohabiting	3.20***	2.15***	2.90***	1.89***
	(0.12)	(0.10)	(0.12)	(0.09)
Divorced—not cohabiting	2.31***	2.23***	2.20***	2.12***
	(0.12)	(0.12)	(0.12)	(0.12)
Widowed—not cohabiting	3.41***	3.19***	3.56***	3.32***
	(0.65)	(0.61)	(0.68)	(0.64)
Never married—cohabiting	1.12*	1.03	1.07	1.00
	(0.06)	(0.05)	(0.05)	(0.05)
Divorced/widowed—cohabiting	1.49***	1.46***	1.44***	1.43***
	(0.13)	(0.13)	(0.13)	(0.13)
Other	2.00***	1.89***	1.85***	1.72***
	(0.24)	(0.23)	(0.23)	(0.21)
Childless				
No (ref.)		1.00		1.00
		(.)		(.)
Yes		2.23***		2.55***
		(0.09)		(0.10)
Highest level of education				
Elementary or lower			2.16	2.05***
			(0.10)	(0.09)
Upper secondary (ref.)			1.00	1.00
			(.)	(.)
Higher education less than 2 years			0.93	0.88
			(0.06)	(0.06)
Higher education more than 2 years			0.66***	0.65***
			(0.03)	(0.03)
Doctoral education			0.49**	0.47**
			(0.12)	(0.11)
Missing			4.47***	2.98***
			(0.51)	(0.34)
Income quartile				
0–25			1.38***	1.34***
			(0.05)	(0.05)
26–50 (ref.)			0.69***	1.00
			(0.03)	(.)
51–75			0.64***	0.61***
			(0.03)	(0.03)
76–100			0.64***	0.52***
			(0.04)	(0.03)
Missing			0.00	0.00
			(.)	(.)
*N*	1,274,555	1,274,555	1,274,555	1,274,555
Deaths	4,013	4,013	4,013	4,013

Hazard ratio coefficients; Standard errors in parentheses

* $$p<0.05$$, ** $$p<0.01$$, *** $$p<0.001$$

**Table 14:** Mortality risk by partnership status for Swedish-born women aged 50–64 in Sweden, 2012–2017

	1	1a	1b	2
Partnership status				
Married (ref.)	1.00	1.00	1.00	1.00
	(.)	(.)	(.)	(.)
Never married—not cohabiting	2.60***	2.15***	2.57***	2.14***
	(0.06)	(0.05)	(0.06)	(0.05)
Divorced—not cohabiting	1.88***	1.88***	2.07***	2.05***
	(0.04)	(0.04)	(0.05)	(0.05)
Widowed—not cohabiting	1.63***	1.61***	1.98***	1.94***
	(0.07)	(0.07)	(0.09)	(0.09)
Never married—cohabiting	1.36***	1.26***	1.31***	1.23***
	(0.04)	(0.04)	(0.04)	(0.04)
Divorced/widowed—cohabiting	1.29***	1.29***	1.41***	1.41***
	(0.05)	(0.05)	(0.05)	(0.05)
Other	1.63***	1.58***	1.64***	1.59***
	(0.14)	(0.14)	(0.14)	(0.14)
Childless				
No (ref.)		1.00		1.00
		(.)		(.)
Yes		1.57***		1.53***
		(0.03)		(0.03)
Highest level of education				
Elementary or lower			1.35***	1.33***
			(0.03)	(0.03)
Upper secondary (ref.)			1.00	1.00
			(.)	(.)
Higher education less than 2 years			0.84***	0.82***
			(0.04)	(0.04)
Higher education at least 2 years			0.74***	0.72***
			(0.02)	(0.02)
Doctoral education			0.52***	0.49***
			(0.07)	(0.07)
Missing			2.95***	2.45***
			(0.25)	(0.21)
Income quartile				
0–25			1.86***	1.82***
			(0.04)	(0.04)
26–50 (ref.)			1.00	1.00
			(.)	(.)
51–75			0.71***	0.70***
			(0.02)	(0.02)
76–100			0.75***	0.74***
			(0.02)	(0.02)
Missing			0.00	0.00
			(.)	(.)
*N*	1,090,786	1,090,786	1,090,786	1,090,786
Deaths	15,170	15,170	15,170	15,170

Hazard ratio coefficients; Standard errors in parentheses

* $$p<0.05$$, ** $$p<0.01$$, *** $$p<0.001$$

**Table 15:** Mortality risk by partnership status for Swedish-born women aged 65 and above in Sweden, 2012–2017

	1	1a	1b	2
Partnership status				
Married (ref.)	1.00	1.00	1.00	1.00
	(.)	(.)	(.)	(.)
Never married—not cohabiting	1.53***	1.47***	1.55***	1.47***
	(0.01)	(0.02)	(0.02)	(0.02)
Divorced—not cohabiting	1.45***	1.45***	1.43***	1.43***
	(0.01)	(0.01)	(0.01)	(0.01)
Widowed—not cohabiting	1.21***	1.21***	1.18***	1.18***
	(0.01)	(0.01)	(0.01)	(0.01)
Never married—cohabiting	1.45***	1.41***	1.41***	1.36***
	(0.04)	(0.04)	(0.04)	(0.04)
Divorced/widowed—cohabiting	1.18***	1.17***	1.15***	1.15***
	(0.02)	(0.02)	(0.02)	(0.02)
Other	1.67***	1.67***	1.64***	1.64***
	(0.02)	(0.02)	(0.02)	(0.02)
Childless				
No (ref.)		1.00		1.00
		(.)		(.)
Yes		1.06***		1.09***
		(0.01)		(0.01)
Highest level of education				
Elementary or lower			1.09***	1.10***
			(0.01)	(0.01)
Upper secondary (ref.)			1.00	1.00
			(.)	(.)
Higher education less than 2 years			0.90***	0.89***
			(0.02)	(0.02)
Higher education at least 2 years			0.82***	0.81***
			(0.01)	(0.01)
Doctoral education			0.71***	0.71***
			(0.04)	(0.04)
Missing			0.99	0.99
			(0.02)	(0.02)
Income quartile				
0–25			0.95	0.95***
			(0.01)	(0.01)
26–50			1.00	1.00
			(.)	(.)
51–75			0.87***	0.87***
			(0.01)	(0.01)
76–100			0.87***	0.87***
			(0.01)	(0.01)
Missing			0.00	0.00
			(0.00)	(0.00)
*N*	1,153,737	1,153,737	1,153,737	1,153,737
Deaths	226,235	226,235	226,235	226,235

Hazard ratio coefficients; Standard errors in parentheses

* $$p<0.05$$, ** $$p<0.01$$, *** $$p<0.001$$

## References

[CR1] Agree, E. M., & Glaser, K. (2009). Demography of informal caregiving. In P. Uhlenberg (Ed.), *International handbook of population aging* (pp. 647–668). Springer.

[CR2] Allison, P. (2009). *Fixed effects regression models*. SAGE Publications.

[CR3] Andersson, G., Thomson, E., & Duntava, A. (2017). Life-table representations of family dynamics in the 21st century. *Demographic Research,**37*, 1081–1230.

[CR4] Barclay, K., Keenan, K., Grundy, E., Kolk, M., & Myrskylä, M. (2016). Reproductive history and post-reproductive mortality: A sibling comparison analysis using Swedish register data. *Social Science & Medicine,**155*, 82–92. 26994961 10.1016/j.socscimed.2016.02.043

[CR5] Ben-Shlomo, Y., Smith, G. D., Shipley, M., & Marmot, M. G. (1993). Magnitude and causes of mortality differences between married and unmarried men. *Journal of Epidemiology and Community Health,**47*(3), 200–205. 8350032 10.1136/jech.47.3.200 PMC1059766

[CR6] Billari, F. C., & Liefbroer, A. C. (2010). Towards a new pattern of transition to adulthood? *Advances in Life Course Research,**15*(2–3), 59–75.

[CR7] Bowling, A. (1987). Mortality after bereavement: A review of the literature on survival periods and factors affecting survival. *Social Science & Medicine,**24*(2), 117–124. 3551087 10.1016/0277-9536(87)90244-9

[CR8] Brown Bulanda, J. R., & Lee, G. R. (2012). Transitions into and out of cohabitation in later life. *Journal of Marriage and Family,**74*(4), 774–793. 23226875 10.1111/j.1741-3737.2012.00994.x PMC3516860

[CR9] Brown, S. L., Lin, I.-F., Hammersmith, A. M., & Wright, M. R. (2019). Repartnering following gray divorce: The roles of resources and constraints for women and men. *Demography,**56*(2), 503–523. 30632111 10.1007/s13524-018-0752-x PMC6450723

[CR10] Brown, S. L., & Wright, M. R. (2017). Marriage, cohabitation, and divorce in later life. *Innovation in Aging,**1*(2), igx015. 30480114 10.1093/geroni/igx015 PMC6177067

[CR11] Carlsson, A. C., Starrin, B., Gigante, B., Leander, K., Hellenius, M.-L., & de Faire, U. (2014). Financial stress in late adulthood and diverse risks of incident cardiovascular disease and all-cause mortality in women and men. *BMC Public Health,**14*(1), 17. 24406139 10.1186/1471-2458-14-17 PMC3931669

[CR12] Carr, D., & Springer, K. W. (2010). Advances in families and health research in the 21st century. *Journal of Marriage and the Family,**72*(3), 743–761.

[CR13] Chudnovskaya, M. (2019). Trends in childlessness among highly educated men in Sweden. *European Journal of Population,**35*(5), 939–958. 31832031 10.1007/s10680-018-9511-3 PMC6883008

[CR14] Cox, D. R. (1972). Regression models and life-tables. *Journal of the Royal Statistical Society, Series B: Statistical Methodology,**34*(2), 187–220.

[CR15] Drefahl, S. (2012). Do the married really live longer? The role of cohabitation and socioeconomic status. *Journal of Marriage and Family,**74*(3), 462–475.

[CR16] Drefahl, S., Lundström, H., Modig, K., & Ahlbom, A. (2012). The era of centenarians: Mortality of the oldest old in Sweden. *Journal of Internal Medicine,**272*(1), 100–102. 22243214 10.1111/j.1365-2796.2012.02518.x

[CR17] Drefahl, S., & Mussino, E. (2023). How does the age of the youngest child affect parental survival? *Genus,**79*(1), 10.

[CR18] Dupre, M. E., Beck, A. N., & Meadows, S. O. (2009). Marital trajectories and mortality among US adults. *American Journal of Epidemiology,**170*(5), 546–555. 19584130 10.1093/aje/kwp194 PMC2732990

[CR19] Dykstra, P. A. (2009). Childless old age. In P. Uhlenberg (Ed.), *International handbook of population aging,* (pp. 671–690). Springer.

[CR20] Franke, S., & Kulu, H. (2018). Mortality differences by partnership status in England and Wales: The effect of living arrangements or health selection? *European Journal of Population,**34*(1), 87–118. 30976244 10.1007/s10680-017-9423-7 PMC6241022

[CR21] Goldman, N. (2001). Social inequalities in health - disentangling the underlying mechanisms. In M. Weinstein, A. I. Hermalin, & M. A. Stoto (Eds.), *Population health and aging: Strengthening the dialogue between epidemiology and demography, annals of the New York Academy of Sciences* (Vol. 954, pp. 118–139). New York Academy of Sciences. 11797854

[CR22] Goldman, N. (2015). Mortality differentials: Selection and causation. In J. D. Wright (Ed.), *International encyclopedia of the social & behavioral sciences* (2nd ed., pp. 851–856). Elsevier.

[CR23] Grundy, E. M., & Tomassini, C. (2010). Marital history, health and mortality among older men and women in England and Wales. *BMC Public Health,**10*(1), 554. 10.1186/1471-2458-10-554 PMC2954998 20843303

[CR24] Hemström, Ö. (1996). Is marriage dissolution linked to differences in mortality risks for men and women? *Journal of Marriage and Family,**58*(2), 366–378.

[CR25] Heuveline, P., & Timberlake, J. M. (2004). The role of cohabitation in family formation: The United States in comparative perspective. *Journal of Marriage and Family,**66*(5), 1214–1230. 24563549 10.1111/j.0022-2445.2004.00088.x PMC3928685

[CR26] Hoem, J. M. (1997). Educational gradients in divorce risks in Sweden in recent decades. *Population Studies,**51*(1), 19–27.

[CR27] Hu Goldman, N. (1990). Mortality differentials by marital status: An international comparison. *Demography,**27*(2), 233–250. 2332088

[CR28] Hu, Y., Leinonen, T., van Hedel, K., Myrskylä, M., & Martikainen, P. (2019). The relationship between living arrangements and higher use of hospital care at middle and older ages: To what extent do observed and unobserved individual characteristics explain this association? *BMC Public Health,**19*(1), 1011. 31357984 10.1186/s12889-019-7296-x PMC6664712

[CR29] Jónsson, A. K. (2021). A nation of bastards? Registered cohabitation, childbearing, and first-marriage formation in Iceland, 1994–2013. *European Journal of Population,**37*(1), 65–95. 33597836 10.1007/s10680-020-09560-2 PMC7865053

[CR30] Kolk, M., & Barclay, K. (2021). Do income and marriage mediate the relationship between cognitive ability and fertility? Data from Swedish taxation and conscriptions registers for men born 1951–1967. *Intelligence,**84*, 101514.

[CR31] Koskinen, S., Joutsenniemi, K., Martelin, T., & Martikainen, P. (2007). Mortality differences according to living arrangements. *International Journal of Epidemiology,**36*(6), 1255–1264. 17971389 10.1093/ije/dym212

[CR32] Lesthaeghe, R. (1995). The second demographic transition in western countries: An interpretation. In K. Oppenheim Mason & A.-M. Jensen (Eds.), *Gender and family change in industrialized countries* (pp. 17–62). Oxford University Press.

[CR33] Lillard, L. A., & Panis, C. W. A. (1996). Marital status and mortality: The role of health. *Demography,**33*(3), 313–327. 8875065

[CR34] Lindström, M., Pirouzifard, M., Rosvall, M., & Fridh, M. (2024). Marital status and cause-specific mortality: A population-based prospective cohort study in southern Sweden. *Preventive Medicine Reports,**37*, 102542. 38169998 10.1016/j.pmedr.2023.102542 PMC10758969

[CR35] Manzoli, L., Villari, P., Pirone, G. M., & Boccia, A. (2007). Marital status and mortality in the elderly: A systematic review and meta-analysis. *Social Science & Medicine,**64*(1), 77–94. 17011690 10.1016/j.socscimed.2006.08.031

[CR36] Murphy, M., Grundy, E., & Kalogirou, S. (2007). The increase in marital status differences in mortality up to the oldest age in seven European countries, 1990–99. *Population Studies,**61*(3), 287–298. 17979003 10.1080/00324720701524466

[CR37] Ohlsson-Wijk, S. (2011). Sweden’s marriage revival: An analysis of the new-millennium switch from long-term decline to increasing popularity. *Population Studies,**65*(2), 183–200. 21630166 10.1080/00324728.2011.574724

[CR38] Ohlsson-Wijk, S., Turunen, J., & Andersson, G. (2020). Family forerunners? An overview of family demographic change in Sweden. In D. N. Farris & A. J. J. Bourque (Eds.), *International handbook on the demography of marriage and the family* (Vol. 7, pp. 65–77). Springer.

[CR39] Olah, L., Karlsson, L., & Sandström, G. (2020). Committed to independence? An exploratory study of living-apart-together (LAT) in contemporary Sweden.

[CR40] Oláh, L. S., & Bernhardt, E. (2008). Sweden: Combining childbearing and gender equality. *Demographic Research,**19*, 1105–1144.

[CR41] Perelli-Harris, B., Sigle-Rushton, W., Kreyenfeld, M., Lappegård, T., Keizer, R., & Berghammer, C. (2010). The educational gradient of childbearing within cohabitation in Europe. *Population and Development Review,**36*(4), 775–801. 21174870 10.1111/j.1728-4457.2010.00357.x

[CR42] Rapp, I. (2018). Partnership formation in young and older age. *Journal of Family Issues,**39*(13), 3363–3390.

[CR43] Rendall, M. S., Weden, M. M., Favreault, M. M., & Waldron, H. (2011). The protective effect of marriage for survival: A review and update. *Demography,**48*(2), 481–506. 21526396 10.1007/s13524-011-0032-5

[CR44] Roelfs, D. J., Shor, E., Kalish, R., & Yogev, T. (2011). The rising relative risk of mortality for singles: Meta-analysis and meta-regression. *American Journal of Epidemiology,**174*(4), 379–389. 21715646 10.1093/aje/kwr111

[CR45] Sassler, S., & Lichter, D. T. (2020). Cohabitation and marriage: Complexity and diversity in union-formation patterns. *Journal of Marriage and Family,**82*(1), 35–61.

[CR46] Scafato, E., Galluzo, L., Gandin, C., Ghirini, S., Baldereschi, M., Capurso, A., Maggi, S., & Farchi, G. (2008). Marital and cohabitation status as predictors of mortality: A 10-year follow-up of an Italian elderly cohort. *Social Science & Medicine,**67*(9), 1456–1464. 18675500 10.1016/j.socscimed.2008.06.026

[CR47] Sobotka, T. (2008). Overview Chapter 6: The diverse faces of the second demographic transition in Europe. *Demographic Research,**19*, 171–224.

[CR48] Sobotka, T., & Toulemon, L. (2008). Overview Chapter 4: Changing family and partnership behaviour: Common trends and persistent diversity across Europe. *Demographic Research,**19*, 85–138.

[CR49] Staehelin, K., Schindler, C., Spoerri, A., Zemp Stutz, E., for the Swiss National Cohort Study Group. (2012). Marital status, living arrangement and mortality: Does the association vary by gender? *Journal of Epidemiology and Community Health,**66*(7), e22–e22. 22012962 10.1136/jech.2010.128397

[CR50] Statistics Sweden (2017). Registerbaserad hushållsstatistik.

[CR51] Thomson, E. (2014). Family complexity in Europe. *The ANNALS of the American Academy of Political and Social Science,**654*(1), 245–258. 10.1177/0002716214524515 PMC4200481 25332509

[CR52] Thomson, E., Winkler-Dworak, M., & Beaujouan, É. (2019). Contribution of the rise in cohabiting parenthood to family instability: Cohort change in Italy, Great Britain, and Scandinavia. *Demography,**56*(6), 2063–2082. 31713128 10.1007/s13524-019-00823-0 PMC6915116

[CR53] Umberson, D. (1987). Family status and health behaviors: Social control as a dimension of social integration. *Journal of Health and Social Behavior,**28*(3), 306–319. 3680922

[CR54] van Hedel, K., Martikainen, P., Moustgaard, H., & Myrskylä, M. (2018). Cohabitation and mental health: Is psychotropic medication use more common in cohabitation than marriage? *SSM - Population Health,**4*, 244–253. 29854908 10.1016/j.ssmph.2018.01.001 PMC5976833

[CR55] Vespa, J. (2012). Union formation in later life: Economic determinants of cohabitation and remarriage among older adults. *Demography,**49*(3), 1103–1125. 22549155 10.1007/s13524-012-0102-3

[CR56] Waldron, I., Hughes, M. E., & Brooks, T. L. (1996). Marriage protection and marriage selection—prospective evidence for reciprocal effects of marital status and health. *Social Science & Medicine,**43*(1), 113–123. 8816016 10.1016/0277-9536(95)00347-9

[CR57] World Values Survey. (2020). *The Inglehart-Welzel World Cultural Map—World Values Survey 7 [Provisional version]*. World Values Survey Association: Technical report.

[CR58] Wright, M. R. (2020). Relationship quality among older cohabitors: A comparison to remarrieds. *The Journals of Gerontology: Series B,**75*(8), 1808–1817. 10.1093/geronb/gbz069 31247086

[CR59] Wyke, S., & Ford, G. (1992). Competing explanations for associations between marital-status and health. *Social Science & Medicine,**34*(5), 523–532. 1604359 10.1016/0277-9536(92)90208-8

